# Dosimetric comparison of coplanar and noncoplanar beam arrangements for radiotherapy of patients with lung cancer: A meta‐analysis

**DOI:** 10.1002/acm2.13197

**Published:** 2021-02-26

**Authors:** Min Ma, Wenting Ren, Minghui Li, Chuanmeng Niu, Jianrong Dai

**Affiliations:** ^1^ National Cancer Center/National Clinical Research Center for Cancer/Cancer Hospital Chinese Academy of Medical Sciences and Peking Union Medical College Beijing China

**Keywords:** coplanar beam arrangement, lung cancer, meta‐analysis, noncoplanar

## Abstract

**Purpose:**

Radiotherapy plays an important role in the treatment of lung cancer, and both coplanar beam arrangements (CBA) and noncoplanar beam arrangements (NCBA) are adopted in clinic practice. The aim of this study is to answer the question whether NCBA are dosimetrically superior to CBA.

**Methods:**

Search of publications were performed in PubMed, Web of Science, and the Cochran Library till March 2020. The searching terms were as following: ((noncoplanar) or ("non coplanar") or ("4pi") or ("4π")) AND (("lung cancer") or ("lung tumor") or ("lung carcinoma")) AND ((radiotherapy) or ("radiation therapy")). The included studies and extracted data were manually screened. All forest and funnel plots were carried out with RevMan software, and the Egger’s regression asymmetry tests were conducted with STATA software.

**Results:**

Nine studies were included and evaluated in the meta‐analysis and treatment plans were designed with both CBA and NCBA. For the planning target volumes (PTV), D98%, D2%, the conformity index (CI), and the gradient index (GI) had no statistically significant difference. For organs‐at‐risk (OAR), V20 of the whole lung and the maximum dose of the spinal cord were significantly reduced in NCBA plans compared with CBA ones. But V10, V5, and mean dose of the whole lung, the maximum dose of the heart, and the maximum dose of the esophagus exhibited no significant difference when the two types of beam arrangements were compared.

**Conclusion:**

After combining multicenter results, NCBA plans have significant advantages in reducing V20 of the whole lung and max dose of spinal cord.

## 1 INTRODUCTION

The incidence and mortality of lung cancer are the highest in a variety of malignancies, and the incidence of lung cancer grows every year.^1^ By 2020, approximately 228 820 lung cancer patients are expected to be diagnosed in the United States (US), and 135 720 of these patients may die from the disease.^2^ Currently, the preferred treatment for lung cancer is surgery. Moreover, radiotherapy plays a more and more important role in the treatment of lung cancer if the patient is unable to be treated surgically. In particular, stereotactic body radiation therapy (SBRT) is a method that can be used for treating inoperable, nonsmall cell lung cancers (NSCLC) and lung metastases.^3^ According to recent studies,^4^, ^5^, ^6^, ^7^, ^8^ SBRT has exhibited favorable clinical outcomes. To achieve a high local control rate and a low toxicity, SBRT treatment of the lung cancer has been performed on gantry‐mounted linear accelerators (LINACs) using noncoplanar or coplanar three‐dimensional conformal radiation therapy (3DCRT),^9^, ^10^ intensity modulated radiation therapy (IMRT),^10^, ^11^ and volumetric modulated arc therapy (VMAT)^12^, ^13^, ^14^ to acquire optimal dose distributions^15^, ^16^, ^17^, ^18^ and increase the efficiency of treatment delivery.

Several studies^19^, ^20^, ^21^, ^22^, ^23^, ^24^, ^25^, ^26^, ^27^, ^28^ have compared coplanar beam arrangements (CBA) and noncoplanar beam arrangements (NCBA) for the radiotherapy of lung cancer, which is delivered on gantry‐mounted LINACs. However, whether NCBA are superior to CBA remains unclear. Thus, we performed this meta‐analysis to compare CBA and NCBA in terms of D98%, D2%, the conformity index (CI), and the gradient index (GI) of the planning target volume (PTV) and organs‐at‐risk (OAR) sparing, including the whole lung, the spinal cord, the heart, and the esophagus.

## 2 MATERIAL AND METHODS

### 2.A Search strategy

Systematic searches were conducted in PubMed, Web of Science, and the Cochrane Library. The searching terms were as following: ((noncoplanar) or ("non coplanar") or ("4pi") or ("4π")) AND (("lung cancer") or ("lung tumor") or ("lung carcinoma")) AND ((radiotherapy) or ("radiation therapy")). No language restrictions were imposed. Any disagreements or discrepancies were resolved by consensus. The searching strategy of Web of Science was taking as an example: ((noncoplanar) or ("non coplanar") or ("4pi") or ("4π") [theme]) AND (("lung cancer") or ("lung tumor") or ("lung carcinoma") [theme]) AND ((radiotherapy) or ("radiation therapy") [theme]).

### 2.B Inclusion and exclusion criteria

A study was included in this meta‐analysis if it fulfilled four predefined criteria: (a) A study was selected if it provides dose information or assessment of lung cancer patients who had been treated with coplanar compared with noncoplanar beams; (b) Coplanar and noncoplanar radiotherapy plans for each patient were designed; (c) Sufficient dosimetric data were contained in the study; and (d) Studies were published up to March 2020.

A study was excluded, even if satisfying the above inclusion criteria, if it (a) investigated clinical trial and clinical outcomes; (b) was not using photon radiotherapy; (c) provided data lacked mean or standard deviation; and (d) was not a scientific paper (e.g., conference abstract, conference proceeding, book, patent).

### 2.C Data extraction

Information extracted from each article included: first author, year of publication, sample size, prescribed dose, number of fractions, PTV (D98%, D2%, CI and GI), the whole lung (mean dose, V10, V5, and V20), the spinal cord (maximum dose), the heart (maximum dose), and the esophagus (maximum dose). If VMAT, N‐VMAT, and N‐FFF‐VMAT are emerged in the article, VMAT and N‐VMAT would be chosen. When there is only the maximum dose of PTV, D2% of PTV is replace with the maximum dose. Similarly, when there is only the minimum dose of PTV, D98% of PTV is taken place of the minimum dose.

### 2.D Statistical analysis

The meta‐analysis and statistics included forest plots, and publication bias. Visual inspection of the funnel plot as well as the Egger’s regression asymmetry test was applied to assess publication bias. All forest and funnel plots were performed using RevMan (Version 5.3; Cochrane Collaboration, Oxford, UK) and the Egger’s regression asymmetry tests were conducted with STATA software (Version 16.0; Stata Corporation, College Station, TX). Generic inverse variance method^29^ was used to calculate the standardized mean difference (SMD).^30^, ^31^ The heterogeneity of all included studies was evaluated by calculating the $I^{2}$statistic.^31^ A fixed‐effects model was applied when the $I^{2}$statistic < 50%, indicating that all included studies exhibited homogeneity.^32^ Otherwise, a random‐effects model was applied when the $I^{2}$statistic > 50%.^32^ A *P* < 0.05 was considered as statistically significant.^33^ For the Egger test, *P* > 0.1 was considered as no publication bias.^34^

## 3 RESULTS

### 3.A Study selection and features of the included studies

**Fig. 1:**
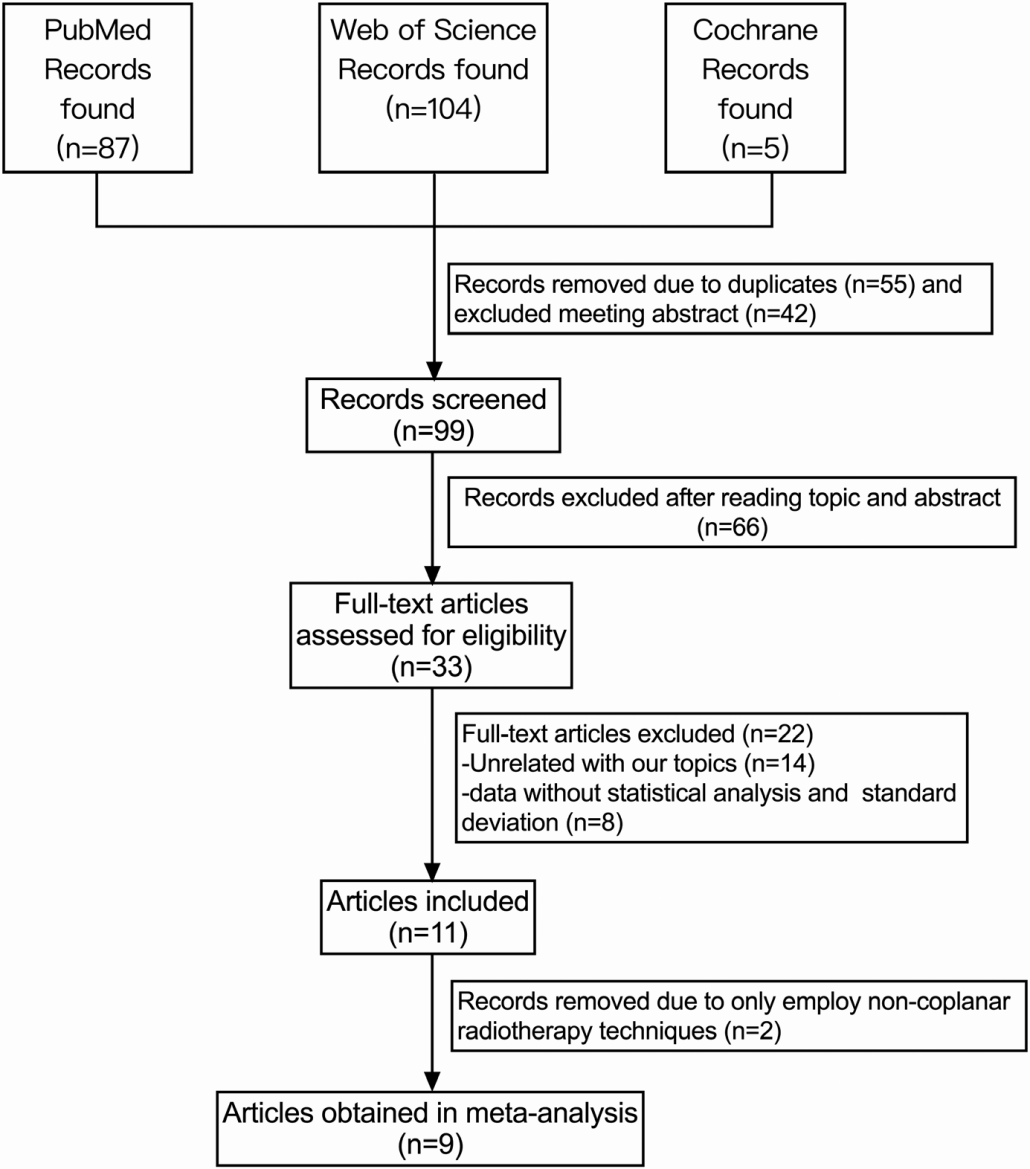
Flow chart detailing the search strategy and identification of studies.

The total numbers of relevant studies recorded during the initial searches from PubMed, Web of Science, and the Cochrane Library were 87, 104, and 5, respectively. After elimination of duplicates and conference abstracts, 99 articles were identified. After the topics and abstracts were read, 66 additional papers were excluded because they were not pertinent to the subject matter. Twenty‐two studies for dosimetric comparisons were also eliminated because 14 studies did not relate to our topics, whereas eight other studies did not record the means or standard deviations. In addition, two studies were excluded because they only compared different noncoplanar radiotherapy techniques. Finally, nine full‐text records were included in the meta‐analysis, and all used SBRT. Flow chart detailing the search strategy and identification of studies was depicted in Fig. 1. The main features of the studies for dosimetric comparison were summarized in Table 1.

**Table 1:** Main features of the studies for dosimetric comparison.

Study	Type	Dose /fr	Patients		PTV				OAR				Dose algorithm
			Coplanar	noncoplanar	D98%	D2%	CI	GI	Whole lung	Spinal cord	Heart	Esophagus	
Fleckenstein 2018^22^	NSCLC	60 Gy/5fr	46	46	Yes	Yes			Mean dose, V10, V5				Monaco, Monte Carlo
Herbert 2013^23^	NSCLC	48 Gy/4fr	10	10		Yes		Yes	Mean dose		Max dose	Max dose	Eclipse, AAA
Ishii 2016^18^	NSCLC	70 Gy/10fr	15	15	Yes	Yes	Yes		Mean dose, V20, V10, V5	Max dose	Max dose	Max dose	Eclipse, AAA
Kim 2019^24^	NSCLC	60 Gy/4fr	20	20	Yes	Yes	Yes	Yes	Mean dose, V20, V10, V5	Max dose	Max dose		Eclipse, AAA
Marnitz 2002^9^	NSCLC	50 Gy/2fr	10	10					Mean dose, V20	Max dose		Max dose	Helios, conjugate gradient algorithm
Tajaldeen 2019^26^	NSCLC	26 Gy/1fr	12	12	Yes	Yes	Yes	Yes	V20, V5	Max dose	Max dose	Max dose	Eclipse, AAA
Tajima 2015^27^	NSCLC	75 Gy/30fr	21	21			Yes		Mean dose, V20, V10, V5	Max dose	Max dose	Max dose	Xio, superposition algorithm
Zhang 2011^14^	early stage	50 Gy/5fr	15	15			Yes	Yes	Mean dose, V20, V5				Pinnacle, DMPO
Ong 2010^13^	NSCLC	18 Gy/3fr, 11 Gy/5fr, 7.5 Gy/8fr	18	18		Yes			V20, V5	Max dose			Eclipse, AAA

Abbreviation: NSCLC = nonsmall cell lung cancer, D98% = dose received by at least 98% volume of the planning target volume, D2% = dose received by at least 2% volume of the planning target volume, CI = the conformity index, GI = the gradient index, OAR = organs‐at‐risk, V5 = volume of lung receiving 5 Gy or more, V10 = volume of lung receiving 10 Gy or more, V20 = volume of lung receiving 20 Gy or more, AAA = anisotropic analytic algorithm, DMPO = direct machine parameters optimization.

### 3.B Dosimetric comparisons of coplanar and noncoplanar beam arrangements for the radiotherapy of lung cancer

**Fig. 2:**
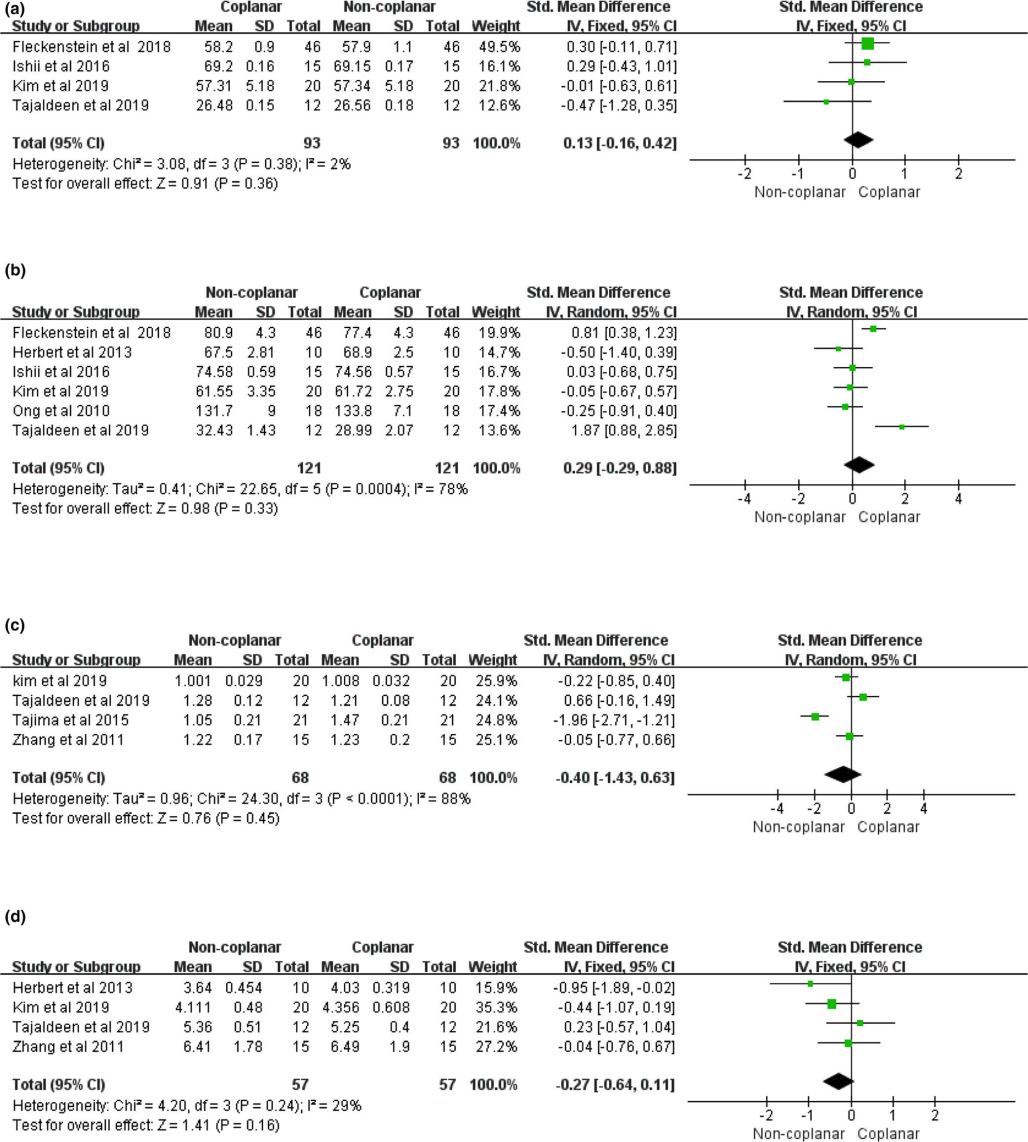
Forest plot of (a) D98%, (b) D2%, (c) CI, and (d) GI of PTV between CBA and NCBA plans.

D98%, D2%, CI and GI of PTV did not show any significant differences between the two types of beam arrangements with mean differences of 0.13 (95% confidence interval (95% CI) [−0.16, 0.42], $I^{2}$: 2%, *P* = 0.36) [Fig. 2(a)], 0.29 (95% CI [−0.29, 0.88], $I^{2}$: 78%, *P* = 0.33) [Fig. 2(b)]; −0.40 (95% CI [−1.43, 0.63], $I^{2}$: 88%, *P* = 0.45) [Fig. 2(c)], and −0.27 (95% CI [−0.64, 0.11], $I^{2}$: 29%, *P* = 0.16) [Fig. 2(d)] respectively.

**Fig. 3:**
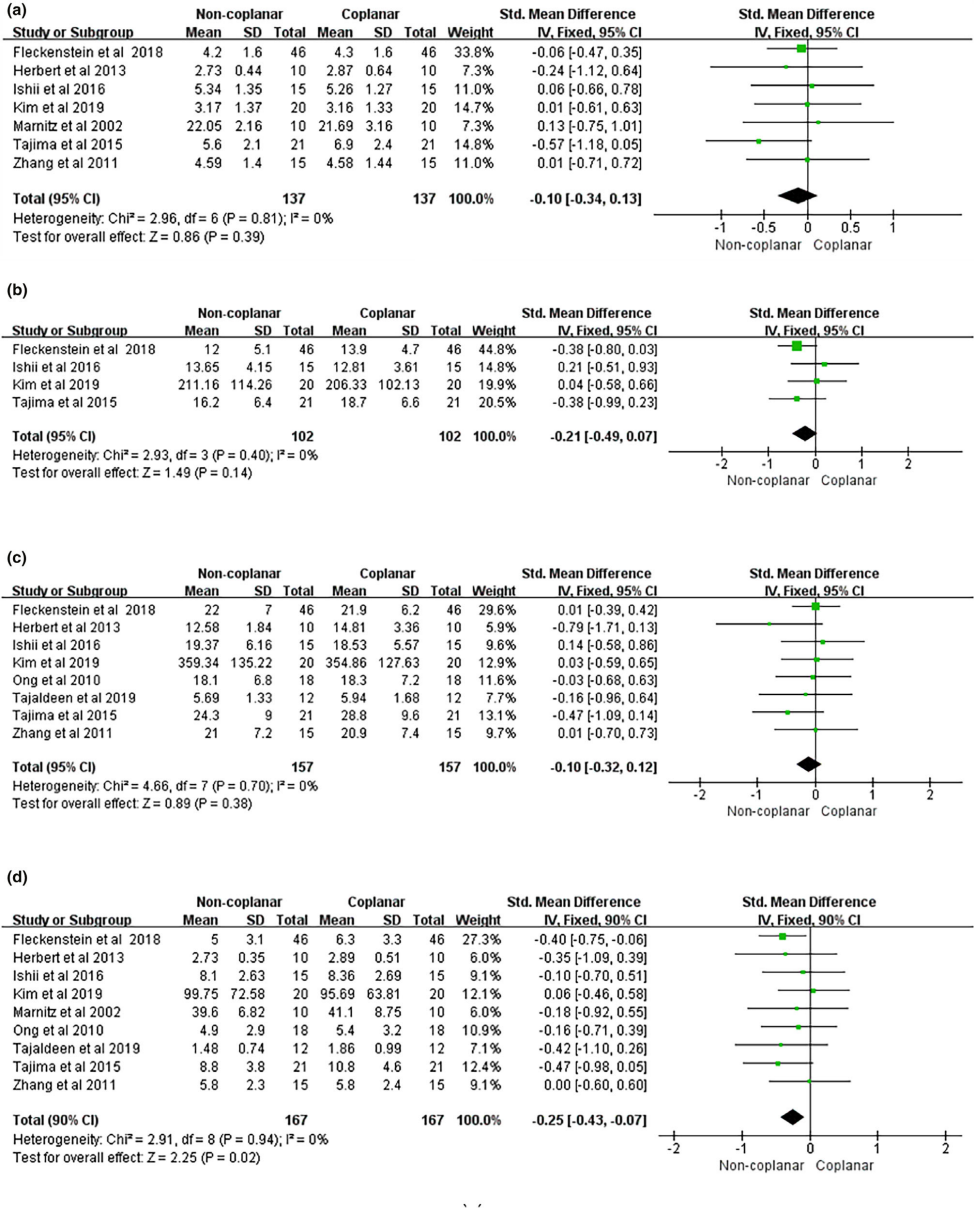
Forest plot of (a) mean dose, (b) V10, (c) V5, and (d) V20 of whole lung between CBA and NCBA plans.

**Fig. 4:**
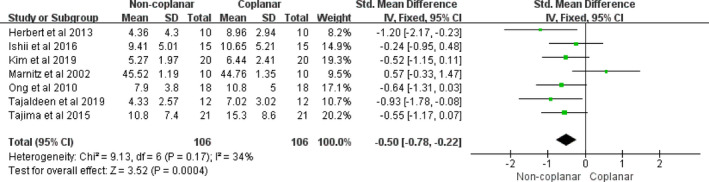
Forest plot of maximum dose in spinal cord between CBA and NCBA plans.

**Fig. 5:**
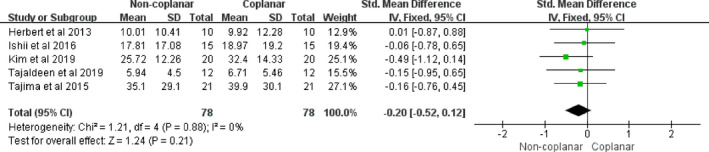
Forest plot of maximum dose in heart between CBA and NCBA plans.

**Fig. 6:**
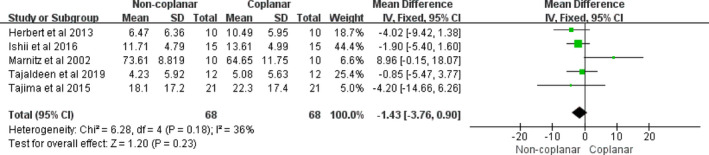
Forest plot of maximum dose in esophagus between CBA and NCBA plans.

The whole lung, the spinal cord, the heart, and the esophagus were the four main OARs in the radiotherapy treatment of lung cancer. In the case of the whole lung, the mean dose, V10, and V5 did not contribute to any differences between CBA and NCBA, with a mean difference of −0.10 (95% CI [−0.34, 0.13], $I^{2}$: 0%, *P* = 0.39) [Fig. 3(a)], –0.21 (95% CI [−0.49, 0.07], $I^{2}$: 0%, *P* = 0.14) [Fig. 3(b)], and −0.10 (95% CI [−0.32, 0.12], $I^{2}$: 0%, *P* = 0.38) [Fig. 3(c)], respectively. However, the V20 of the whole lung had significantly decreased for NCBA than CBA for radiotherapy (mean volume difference: −0.25, 95% CI [−0.43, −0.07], $I^{2}$: 0%, *P* = 0.02) [Fig. 3(d)]. For the spinal cord, the maximum dose of NCBA was significantly lower than that of CBA for radiotherapy, with a mean maximum dose difference of −0.50 (95% CI [−0.78, −0.22], $I^{2}$: 34%, *P* = 0.0004) (Fig. 4). The maximum doses in the heart and the esophagus did not show statistical differences between the two beam arrangements techniques, with mean maximum dose differences of −0.20 (95% CI [−0.52, 0.12], $I^{2}$: 0%, *P* = 0.21) (Fig. 5), and −1.43 (95% CI [−3.76, 0.90]; $I^{2}$: 36%, *P* = 0.23) (Fig. 6).

### 3.C Publication bias

**Fig. 7:**
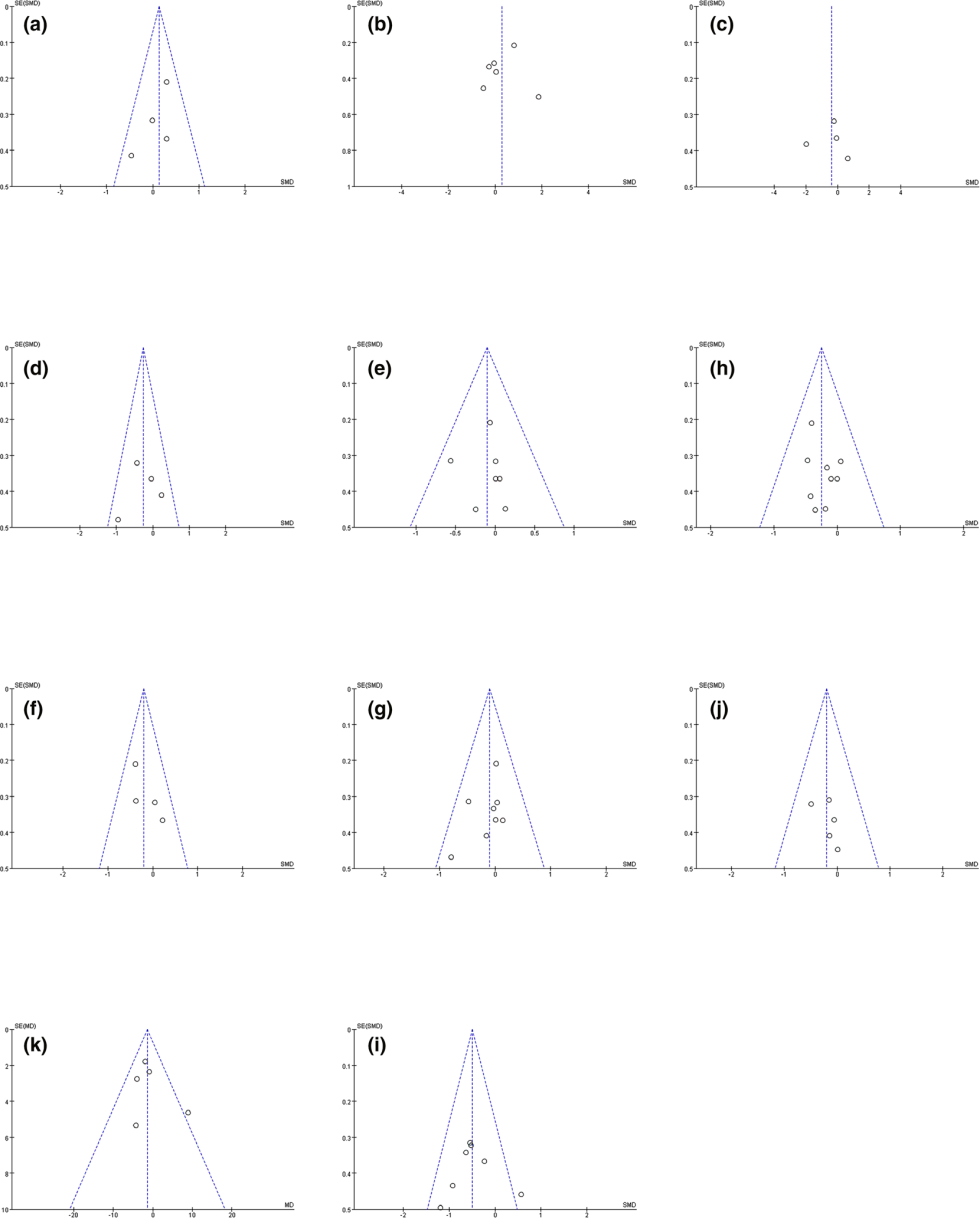
Funnel plots for publication bias of selected meta‐analysis. (a) D98%, (b) D2%, (c) CI, (d) GI, (e) mean dose of the whole lung, (f) V10 of the whole lung, (g) V5 of the whole lung, (h) V20 of the whole lung, (i) maximum dose of spinal cord, (j) maximum dose of heart, (k) maximum dose of esophagus.

The publication bias for the meta‐analysis was difficult to estimate because of the limited number of included studies. The funnel plots seemed to be symmetrical on visual inspection of D98%, GI, V20, and V10 of whole lung (Fig. 7). In addition, Egger’s tests were applied, which demonstrated no publication bias for all the parameters (D98%: *P* = 0.286, D2%: *P* = 0.726, CI: *P* = 0.917, GI: *P* = 0.771, mean dose of the whole lung: *P *= 0.919, V20 of the whole lung: *P* = 0.404, V10 of the whole lung: *P* = 0.219, V5 of the whole lung: *P* = 0.273, maximum dose of the heart: *P* = 0.299, maximum dose of the spinal cord: *P* = 0.995, and maximum dose of the esophagus: *P* = 0.561, respectively). All funnel plots were exhibited in Fig. 7.

## 4 DISCUSSION

For the target dose in CBA and NCBA plans, there is no significant difference in CI, GI, D98%, and D2%. This shows that the coverage of the target dose between CBA and NCBA plans is similar and will not affect the target dose due to different beam arrangements.

The D98% of PTV is considered as the near‐minimum absorbed dose.^35^ The meta‐analysis of the D98% of the PTV has no significant difference (*P* = 0.36) between CBA and NCBA plans. As can be seen from the table on the left in Fig. 2(a), only Tajaldeen et al.^26^ reported that D98% of CBA was higher than that of NCBA. It is known from the included literature that certain beam angles could be limited in order to avoid collisions when we design NCBA plans.^36^

The D2% of the PTV is the near‐maximum absorbed dose.^35^ The meta‐analysis of the D2% of the PTV (*P* = 0.33) has no significant difference between CBA and NCBA plans. Judging which technique is more advantageous is difficult. In addition, the result is heterogeneous. Sensitivity or pressure group analyses need to be conducted for exploring the source of heterogeneity. Additional explorations are beyond the purpose of this article.

Conformity index is a complementary tool that defined a score for each treatment plan to allow comparisons of different treatment plans for the same patient.^37^ Five articles were included regarding CI. The included studies used the same definition of conformity index. The meta‐analysis of CI has no significant difference (*P* = 0.45) between CBA and NCBA plans. This may be attributed to the different calculation approaches or to the limited number of articles. From the formula, the closer of the CI value is to 1, the better is the conformity of PTV.^38^ Since the CI values in these studies are always greater than 1, the smaller CI indicated better target conformality. In addition, the CI has a higher heterogeneous result because of the small sample size, planning strategies, or optimization algorithm.^39^

Gradient index is a measure of steep dose gradient outside the target volume.^38^ It is an important index for plan comparison. The meta‐analysis of GI has no significant difference (*P* = 0.16) between CBA and NCBA plans. Three out of four of studies^14^, ^23^, ^24^ reported that the noncoplanar plans resulted in better GI indices than the coplanar plans for the lung cancer. As can be seen from the table on the left in Fig. 2(d), only Tajaldeen et al.^26^ reported that VMAT resulted in the better than noncoplanar IMRT, without considering the confidence interval. It is known from the included literature that the angle separation of the noncoplanar arc is limited when we design NCBA plans.^14^

The whole lung is the foremost OAR in lung cancer. The mean dose, V20, V10, and V5, are compared in the cases of NCBA and CBA for radiotherapy treatment. Tajima et al.^27^ reported that NCBA improved the plan quality with respect to the whole lung sparing than CBA at the mean dose, V20, V10, and V5. However, Kim et al.^24^ suggested that there was no statistical significance reduction between NCBA and CBA about the whole lung sparing. After the meta‐analysis, it is found that only the V20 of the whole lung is significant (*P* = 0.02). One important dose‐limiting toxicity in lung cancer is radiation pneumonitis, whose occurrence and severity correlates well with V20.^40^ The lower the V20, the lower the risk of radiation pneumonitis.^41^, ^42^, ^43^, ^44^ Therefore, noncoplanar radiotherapy was found to have lower the risk of radiation pneumonitis than coplanar one.

For the spinal cord, a total of six studies were used for the meta‐analysis. This shows that the maximum dose of the spinal cord was significantly decreased (*P* = 0.0004) in NCBA plans. With the exception of Marnitz et al.^9^ who compared noncoplanar 3DCRT and IMRT, others^13^, ^18^, ^24^, ^26^, ^27^ reported that noncoplanar radiotherapy was more advantageous than coplanar radiotherapy on spinal cord sparing. Marnitz et al.^9^ concluded that IMRT can better spare spinal cord compared with N‐3DCRT, which could be caused by the advanced algorithm of inverse optimization using IMRT. In additional, some articles^45^, ^46^, ^47^ considered that high spinal cord dose could increase the risk of developing metastatic spinal cord compression (MSCC) resulting in disability in mobility and poorer quality of life for patients. Finally, it is concluded based on our analysis that noncoplanar radiotherapy has significant advantages in protecting the spinal cord.

The heart is another important OAR. Because the data number of the same dosimetric parameter is limited, only the maximum dose of the heart was discussed. Five articles were included. The maximum dose of the heart has no significant difference (*P* = 0.21) in the cases of NCBA and CBA plans. This indicates that the maximum heart dose may be influenced in a complex manner by the planning methods, such as the couch angles and constraints due to target shape and location. In this way, it is difficult to reflect the advantages of noncoplanar radiotherapy in protecting the heart.

As can be seen from the table on the left in Fig. 6, the four included literatures showed that NCBA radiotherapy plans had a lower esophagus maximum dose than CBA ones. No significant difference (*P* = 0.23) in the maximum dose of esophagus is observed between NCBA and CBA plans. Marnitz et al.^9^ demonstrated that 3DCRT was superior to noncoplanar IMRT in esophagus sparing. This could be attributed to the dose escalation in the IMRT plans, but the dose to the esophagus reduced only in two out of 10 patients.

The other dosimetric parameters of the OARs were not subjected to meta‐analyses mainly because the data number was less than four. Hence, the meta‐analysis does not have much relevance. In this study, Fleckenstein et al.,^22^ Herbert et al.,^23^ Ishii et al.,^18^ Kim et al.^24^ and Ong et al.^13^ all discussed that NCBA plans generally requires more time to deliver the treatment than CBA plans due to the time required for positioning of the couch or involving a large number of beams optimization.^18^, ^25^, ^48^, ^49^ The meta‐analysis of treatment time is not performed mainly because only two articles^22^, ^23^ provided the mean or standard deviation of treatment time. Due to the limited number of studies using noncoplanar treatment methods, the meta‐analysis to only one configuration of treatment planning systems (TPS) and calculation algorithms was restricted.

However, there exists some problems in NCBA radiotherapy such as: (a) collision between the patient (or couch) and the gantry head, (b) longer treatment time than that of the coplanar radiotherapy plans, (c) potentially large patient setup uncertainty by couch rotation, (d) hardware challenge of delivery accuracy and automation, (e) the lack of mature beam orientation optimization programs operating in the noncoplanar space. These limitations lead to restrictions on the design of NCBA plans.

## 5 CONCLUSION

After combining multicenter results, NCBA plans have significant advantages in reducing V20 of the whole lung and max dose of spinal cord. It is suggested to consider NCBA when CBA cannot meet clinical requirements.
